# Impact of specific serotonin receptor modulation on restricted repetitive behaviors

**DOI:** 10.3389/fnbeh.2022.1078983

**Published:** 2022-12-23

**Authors:** Bryan D. Alvarez, Cassandra Cavazos, Cheyenne A. Morales, Shannon M. Lopez, Dionisio A. Amodeo

**Affiliations:** ^1^Department of Psychology, California State University, San Bernardino, San Bernardino, CA, United States; ^2^Department of Neuroscience, Ohio State University, Columbus, OH, United States

**Keywords:** restricted repetitive behaviors, autism, serotonin receptor, RRBS, obsessive compulsive disorder

## Abstract

Restricted, repetitive behaviors (RRBs) are commonly divided into two behavioral categories, lower-order and higher-order RRBs. Individuals displaying lower-order motoric RRBs may express repetitive hand flapping behaviors, body rocking back and forth movements, and continuous body spinning. Higher-order RRBs most commonly cover the behavior inflexibility and cognitive rigidity commonly found in disorders such as autism spectrum disorder and obsessive-compulsive disorder. Various neuropsychiatric disorders are plagued by RRBs yet no FDA-approved treatments have been identified. In rodents, lower-order RRBs are commonly measured through various tasks, such as repetitive self-grooming, marble burying, and stereotypic motor behaviors. This review focuses on the effects that modulation of specific serotonin receptors have on lower-order RRBs. Although there is research examining how changes in 5-HT1A, 5-HT1B, 5-HT2A, 5-HT2B, 5-HT2C, 5-HT3, 5-HT6, and 5-HT7 receptor modulation, more research has focused on the 5-HT1A, 5-HT2A, and 5-HT2C receptors. The accumulating data suggest that increasing 5-HT1A activation decreases RRBs while blocking 5-HT1A activation has no effect on RRBs. While there are mixed findings regarding the impact of 5-HT2A modulation on RRBs, the general trend shows mixed effects of 5-HT2A receptor activation RRB expression, whereas blockade generally decreases RRBs. 5-HT2C receptor activation can modulate RRBs in either direction depending on the 5-HT2C drug used, blocking 5-HT2C activation only seems to show therapeutic properties when 5-HT2C activation is already elevated. The other 5-HT receptors have been explored far less but show promise as potential targets for regulating RRBs. Although it is less clear due to the involvement of 5-HT1D, 5-HT1A activation increases RRBs, and blocking 5-HT1A tends to decrease RRBs. 5-HT2B activation could reduce RRBs, while inhibiting 5-HT2B does not impact RRBs. Increasing 5-HT3 has not been shown to affect RRBs. Yet, increases in RRBs have been observed in Htr3a KO mice. 5-HT6 receptor activation can increase RRBs, while blocking 5-HT6 activity tends to decrease RRBs. Lastly, neither increasing or blocking 5-HT7 activity can reduce RRBs. In sum, there is no uniform pattern in whether all specific 5-HT receptors affect RRBs in either direction, instead, there is evidence suggesting that different 5-HT receptors can modulate RRBs in different directions. Further researching the less explored receptors and aiming to understand why these receptors can differently modulate RRBs, may play a key role in developing therapeutics that treat RRBs.

## Introduction

Restricted repetitive behaviors (RRBs) are present in several neuropsychiatric disorders including schizophrenia, obsessive-compulsive disorder, and autism spectrum disorder. RRBs can similarly affect routine activities or interests in some individuals. RRBs can manifest in the first years of life, leading to a substantial negative impact on daily functioning (DSM-5, 2013). These behaviors are commonly grouped into lower-order and higher-order RRBs. Higher-order RRBs are characterized as rigid, goal-oriented behaviors, such as rituals, compulsions, and restricted routines. In addition, higher-order RRBs are often discussed as cognitive or behavioral inflexibility, including insistence on sameness behaviors and restricted, specific patterns of interest (Kanner, 1943; Turner, 1999; Richler et al., 2007). In most instances, distress often follows the alteration of a familiar environment or routine, such as a change in the layout of a familiar room or being taken on a different route to a familiar place. Conversely, lower-order RRBs include repetitive movements, such as motor stereotypies and repetitive self-inflicted actions. Individuals displaying lower-order motoric RRBs may continually flap their hands, rock back and forth, or spin the wheels of a toy car. In rodents, lower-order RRBs are measured through various tasks, such as self-grooming, marble burying, and stereotypic behaviors (i.e., head and body shakes, licking, forepaw tapping, wet-dog shakes, repetitive jumping, etc.). The current review will focus on lower-order repetitive behaviors, with a large portion of the discussed findings measuring self-grooming and marble burying behaviors.

Serotonin (5-HT) is an indolamine that regulates various biological processes including appetite, mood, sleep, cognition, and executive function (Berger et al., 2009; Ray et al., 2011; Meltzer and Roth, 2013). Serotonin can be found in all brain regions that have been linked to the presence of RRBSs, including basal ganglia structures and orbitofrontal cortex (Sears et al., 1999; Herbert et al., 2003; Hollander et al., 2005; Palencia and Ragozzino, 2006; Burguière et al., 2013; Langen et al., 2014). While there have been studies highlighting striatal regions, secondary motor cortex, and frontal cortical regions, there is still a lack of research involving the role of specific neurochemical abnormalities that can lead to RRBs. Understanding which neurochemical systems may lead to RRBs may lead to more effective treatments aimed at attenuating RRBs. Interestingly, there are no FDA-approved treatments for RRBs, and the pathophysiology and function of RRBs are still unclear (Moss et al., 2009). In Alvarez et al. (2021), we reviewed the way in which 5-HT receptor modulation affects higher-order RRBs (i.e., behavioral inflexibility) hoping to help guide the development of new therapeutics for neuropsychiatric disorders afflicted by behavioral inflexibility. This review will focus on the effects that modulation of specific serotonin receptors has on lower-order RRBs (i.e., repetitive sensory motor behaviors). Representative findings are presented in Table 1.

**Table 1:** Effects of specific serotonin receptor modulation on restricted, repetitive behaviors.

Receptor	Activity	RRB	Manipulation	Assay	Reference
5HT1A	↑1A	↑	8-OH-DPAT	Stereotypic Behavior	Khatri et al. (2014)
	↑1A	↑	8-OH-DPAT	Stereotypic Gnawing	Gaggi et al. (1997)
	↑1A	↑	Buspirone	Self Grooming	Cao and Rodgers (1997b)
	↑1A	–	Flesinoxan	Self Grooming	Molewijk et al. (1995)
	↑1A	–	Ipsapirone	Self Grooming	Molewijk et al. (1995)
	↑1A	–	S15535	Marble Burying	Bruins Slot et al. (2008)
	↑1A	–	Buspirone	Marble Burying	Bruins Slot et al. (2008)
	↑1A	–	Buspirone (BTBR)	Marble Burying	Gould et al. (2011)
	↑1A	↓	S20499	self-grooming	Blanchard et al. (1997)
	↑1A	↓	8-OH-DPAT	self-grooming	Blanchard et al. (1997)
	↑1A	↓	Tandospirone (Shank3B)	self-grooming	Dunn et al. (2020)
	↑1A	↓	Buspirone	Marble Burying	Ichimaru et al. (1995)
	↑1A	↓	8-OH-DPAT	Marble Burying	Ichimaru et al. (1995)
	↑1A	↓	8-OH-DPAT	Marble Burying	Egashira et al. (2008)
	↑1A	↓	Buspirone	Marble Burying	Pires et al. (2013)
	↑1A	↓	Tandospirone	Marble Burying	Abe et al. (1998)
	↑1A	↓	MKC242	Marble Burying	Abe et al. (1998)
	↑1A	↓	Buspirone	Marble Burying	Abe et al. (1998)
	↑1A	↓	Buspirone	Marble Burying	Chen et al. (2019)
	↑1A	↓	8-OH-DPAT	Marble Burying	Bruins Slot et al. (2008)
	↓1A	↑	pMPPI	self-grooming	Cao and Rodgers (1997a)
	↓1A	↑	WAY100635	self-grooming	Bagdy et al. (2001)
	↓1A	–	WAY100635	self-grooming	Ho et al. (2016)
	↓1A	–	WAY100635	self-grooming	Jackson et al. (1998)
	↓1A	–	NDL249	self-grooming	Jackson et al. (1998)
	↓1A	–	WAY100635	Marble Burying	Bruins Slot et al. (2008)
	↓1A	–	WAY100635	Marble Burying	Egashira et al. (2008)
	↓1A	–	WAY100635 (pcp-induced)	Marble Burying	Cascio et al. (2015)
	↓1A	–	WAY100635	Marble Burying	Harasawa et al. (2006)
	↓1A	–	WAY100635	Marble Burying	Pires et al. (2013)
	↓1A	–	WAY100635	Marble Burying	Casarotto et al. (2010)
	↓1A	↓	WAY100635 + Citalopram (compared to Citalopram)	Stereotypic Behavior	Khatri et al. (2014)
	↓1A	↓	Alverine citrate	Marble Burying	Gupta et al. (2014)
	↓1A	↓	WAY100635 (fluvoxamine-induced)	Marble Burying	Harasawa et al. (2006)
5HT1B	↑1B	↑	CGS12066B	Stereotypic Behavior	Khatri et al. (2014)
	↑1B	↓	RU24969	self-grooming	Ho et al. (2016)
	↑1B	↓	RU24969	self-grooming	O’Neill and Parameswaran (1997)
	↓1B	↓	GR127935	self-grooming	Ho et al. (2016)
	↓1B	↓	GR127935 + Citalopram (compared to Citalopram)	Stereotypic Behavior	Khatri et al. (2014)
5-HT2A	↑2A	↑	DOI	Stereotypic Behavior	Kozuru et al. (2000)
	↑2A	↑	DOI	Stereotypic Behavior	Hongyan et al. (2017a)
	↑2A	↑	DOI	Stereotypic Behavior	Hongyan et al. (2017b)
	↑2A	↑	DOI	Stereotypic Behavior	Gaggi et al. (1997)
	↑2A	↑	DOI	Stereotypic Behavior	Wettstein et al. (1999)
	↑2A	—	25CN-NBOH	Marble Burying	Tsybko et al. (2020)
	↑2A	—	TCB2	Marble Burying	Tsybko et al. (2020)
	↑2A	—	DOI	Marble Burying	Tsybko et al. (2020)
	↑2A	↓	DOI (SKF38393-induced d1)	self-grooming	Scalzitti et al. (1999)
	↑2A	↓	DOI	Marble Burying	Odland et al. (2021a)
	↑2A	↓	DOI	Marble Burying	Odland et al. (2021b)
	↑2A	↓	25CN-NBOH	Marble Burying	Odland et al. (2021b)
	↓2A	↑	M100907 (BTBR; intra-ofc)	self-grooming	Amodeo et al. (2017)
	↓2A	—	Mianserin (SKF38393-induced d1)	Self-Groomina	Scalzitti et al. (1999)
	↓2A	—	M100907	Marble Burying	Odland et al. (2021a)
	↓2A	—	M100907	Marble Burying	Bruins Slot et al. (2008)
	↓2A	↓	M100907 (BTBR; intra-dorsomedial striatum)	self-grooming	Amodeo et al. (2017)
	↓2A	↓	M100907 (BTBR; high dose)	self-grooming	Amodeo et al. (2016)
	↓2A	↓	MDL100907 (lsd-inducedin WT)	self-grooming	Rodriguiz et al. (2021)
	↓2A	↓	Ritanserin	Marble Buriina	Bruins Slot et al. (2008)
5-HT2B	↑2B	↑	BW723C86 (sc)	self-grooming	Kennett et al. (1997)
	↑2B	—	BW723C86 [intra-right lateral ventricle (icv)]	self-grooming	Kennett et al. (1997)
	↓2B	—	LY266097	Marble Burying	Bruins Slot et al. (2008)
5-HT2C	↑2C	↑	mCPP	self-grooming	Graf et al. (2003)
	↑2C	↑	mCPP [pretreatment SB242084 (dose)]	self-grooming	Graf et al. (2003)
	↑2C	↑	mCPP (pretreatment p-CPA)	self-grooming	Graf et al. (2003)
	↑2C	↑	mCPP	self-grooming	Bagdy et al. (2001)
	↑2C	↑	mCPP (fawn-hooded rats)	self-grooming	Kantor et al. (2000)
	↑2C	↑	mCPP	self-grooming	Reimer et al. (2018)
	↑2C	↑	mCPP	self-grooming	Wright and Rodgers (2014)
	↑2C	↑	mCPP	Marble Burying	Bhutada et al. (2013)
	↑2C	↓	MK212	self-grooming	Halford et al. (1997)
	↑2C	↓	Ro600175	Marble Burying	Martin et al. (1998)
	↑2C	↓	Ro600332	Marble Burying	Martin et al. (1998)
	↓2C	↓	CPD1	Marble Burying	Rodriguez et al. (2017)
	↓2C	↓	Ro600175	Marble Burying	Rodriguez et al. (2017)
	↓2C	—	SB215505	Self-Groomina	Bruins Slot et al. (2008)
	↓2C	—	SB242084	Marble Burying	Odland et al. (2021b)
	↓2C	—	SB242084 (decreased the elevated grooming from M)	Self-Groomina	Graf et al. (2003)
	↓2C	↓	SB242084 (decreased the elevated grooming from M)	self-grooming	Graf et al. (2003)
	↓2C	↓	SB242084	self-grooming	Bagdy et al. (2001)
5-HT3	↑3	—	2-methyl-5-HT (intra-VTA)	self-grooming	Gillies et al. (1996)
	↓3	↑	HTR3A KO mice	self-grooming	Huang et al. (2021)
5-HT6	↑6	↑	WAY208466	self-grooming	Pereira et al. (2015)
	↓6	—	BGC20761 (BTBR)	Repetitive Jumping	Amodeo et al. (2021)
	↓6	↓	BGC20761 (BTBR)	self-grooming	Amodeo et al. (2021)
5-HT7	↑7	↓	(+)-5-FPT	self-grooming	Canal et al. (2015)
	↑7	↓	(+)-5-FPT	Stereotypic Jumping	Canal et al. (2015)
	↓7	↓	5-HT7−/– KO	Marble Burying	Hedlund and Sutcliffe (2007)
	↓7	↓	SB269970 (in both KO and control)	Marble Burying	Hedlund and Sutcliffe (2007)

Key: ↑receptor target, agonist; ↓receptor target, antagonist; ↑, increased rrbs; ↓, decreased rrbs; —, no effect on rrbs.

## 5-Ht1a

Localization studies have found that 5-HT1A receptors are expressed throughout the CNS, while in raphe nuclei these are somatodendritic receptors, but postsynaptic receptors in cortical and limbic areas (Radja et al., 1991; Miquel et al., 1992; Riad et al., 2000). Several studies have shown that 5-HT1A activation modulates RRB expression. Most of these studies suggest increases in 5-HT1A activation leads to reductions in RRBs, while some suggest activation exacerbates RRB expression. Gaggi et al. (1997) and Khatri et al. (2014) both found that 8-OH-DPAT, a 5-HT1A agonist, increased stereotypic behaviors, producing elevated repetitive vertical head movements in Long-Evan rat pups and stereotypic gnawing in Sprague–Dawley rats, respectively. Conversely, two other 5-HT1A agonists, Flesinoxan and Ipsapirone had no effect on self-grooming behavior in Wistar rats (Molewijk et al., 1995). Bruins Slot et al. (2008) and Gould et al. (2011) also show that neither S15535 nor Buspirone impacted marble burying behaviors, even in the BTBR T+tf/J (BTBR) mouse model of ASD. Most of the 5-HT1A related research suggests that increasing activation decreases RRBs. For instance, three different 5-HT1A agonists (S20499, 8-OH-DPAT, and Tandospirone) reduce self-grooming behaviors, measured in Swiss–Webster mice and Shank3 mouse model of ASD (Blanchard et al., 1997; Dunn et al., 2020). Similar results are found during marble burying with other 5-HT1A agonists including Buspiron, 8-OH-DPAT, Tandospirone, and MKC242 (Ichimaru et al., 1995; Abe et al., 1998; Bruins Slot et al., 2008; Egashira et al., 2008; Pires et al., 2013; Chen et al., 2019). These findings are consistent across several strains of mice (ICR, Swiss, and NMRI mice) and even Wistar rats.

Unlike 5-HT1A agonists, 5-HT1A antagonists tend to produce little change in RRB expression, some studies show that 5-HT1A antagonists can increase and decrease RRBs. The 5-HT1A antagonist, pMPPI, increases self-grooming in Swiss Webster mice, and WAY10063 increases self-grooming in Sprague–Dawley rats (Cao and Rodgers, 1997a; Bagdy et al., 2001). Various studies measuring the effect of WAY100635 on marble burying similarly suggest 5-HT1A activation has little effect on RRBs in NMRI, IRC, Swiss, and C57BL/6J mice (Bruins Slot et al., 2008; Egashira et al., 2008; Casarotto et al., 2010; Pires et al., 2013). Likewise, Cascio et al. (2015) found that 5-HT1A blockade did not affect PCP-induced RRBs in Sprague–Dawley rats. Most studies testing 5-HT1A antagonists find that there is no effect on marble burying, and repetitive self-grooming in Sprague–Dawley rats (Jackson et al., 1998). Conversely, Alverine Citrate, a less commonly studied 5-HT1A antagonist, decreased marble burying in Swiss Albino mice, but only in the higher doses of 10, 15, and 20 mg/kg (Gupta et al., 2014). Similar results were also found with WAY100635 treatment on marble burying, self-grooming, and stereotypic behaviors (Harasawa et al., 2006; Khatri et al., 2014; Ho et al., 2016). These results are more difficult to generalize because Khatri et al. (2014) compared rats that received citalopram to those receiving both citalopram and WAY100635. Interestingly, even though Harasawa et al. (2006) found that WAY100635 alone does not affect marble burying, dose-dependent effects were found when WAY100635 was paired with fluvoxamine 30 (mg/kg). Fluvoxamine, an SSRI, decreased marble burying behavior compared to controls. Yet, when pairing 0.1 mg/kg WAY100635 with fluvoxamine, marble burying was significantly decreased compared to fluvoxamine alone. When the high dose of WAY100635 at 1.0 (mg/kg) was paired with fluvoxamine, marble burying was significantly increased compared to fluvoxamine alone suggesting that 5-HT1A blockade alone does not elevate the expression of repetitive behaviors.

Both 5-HT1A agonists 8-OH-DPAT and Buspirone and the 5-HT1A antagonist WAY 100635 are the most widely studied specific 5-HT1A receptor drugs examining RRBs. The data suggest that 8-OH-DPAT and Busprione tend to decrease RRB expression while the agonist WAY100635 does not significantly impact RRBs, tested across various doses, tasks, and rodent strains. On the contrary, there are a few instances where 8-OH-DPAT and WAY100635 present conflicting results. Khatri et al. (2014) find that 8-OH-DPAT increases stereotypic behavior but defines stereotypic behaviors as repetitive vertical head movements. Repetitive vertical head movements are beyond the scope of this review and are similar to head-twitch responses, common behavioral motor measures not typically labeled as RRBs, and are specifically linked to hallucinogenic compounds. Cao and Rodgers (1997b) found that buspirone increased RRBs, instead of decreasing, at 3 (mg/kg), yet found no effect at the low doses (0.1, 0.3, and 1.0 mg/kg). In the BTBR mouse model of ASD, Gould et al. (2011) found that buspirone had no impact on marble burying. Lastly, most of the data involving the 5-HT1A antagonist WAY100635 displayed no effects on RRBs. The one study that showed WAY100635 increases grooming found modest effects, while both of the studies did find that the 5-HT1A agonist decreased marble burying and grooming after SSRI treatment. With all this considered, increasing 5-HT1A activation appears to decrease RRBs, while blocking 5-HT1A activation does not have a reliable impact on RRBs.

## 5-Ht1b

Although 5-HT1B receptors can be found throughout the CNS, they are primarily expressed within the hippocampus, frontal cortex, basal ganglia, and striatum. Not much is known about the impact that 5-HT1B activation or blockade has on RRBs due to the limited number of studies. Although the three available studies have opposing results, they utilized the same 5-HT1B agonists, CGS12066B and RU24969. Khatri et al. (2014) found that repeated CGS12066B treatment increased stereotypic repetitive vertical head movements in Long Evan rats. On the other hand, the 5-HT1B agonist RU24969 has been found to reduce self-grooming in both C57BL/6J mice and Hooded Lister rats (O’Neill and Parameswaran, 1997; Ho et al., 2016).

Ho et al. (2016) and Khatri et al. (2014) also examined the effects of 5-HT1B antagonist, GR127935 on grooming behavior in C57BL/6J mice and Hooded Lister rats respectively. Their findings suggest that blocking 5-HT1B activation can also decrease RRB expression. According to Khatri et al. (2014), rats were pretreated with the SSRI citalopram exhibited increased self-grooming. These elevated self-grooming behaviors induced by citalopram were reduced when treated with GR127935 before testing. Although these drugs (CGS12066B, RU24969, and GR127935) are all highly selective for 5-HT1B receptors, none of them solely target 5-HT1B receptors. Even though the 5-HT1B agonist, CGS12066B, is 17 times more selective for 5-HT1B receptors, it also has low affinity for 5-HT1D receptors (Neale et al., 1987; Middlemiss and Hutson, 1990). The other 5-HT1B agonist mentioned, RU24969, primarily targets 5-HT1B receptors (Ki = 0.38 nmol) but also has low affinity for 5-HT1A receptors (Ki = 2.5 nmol; Peroutka, 1986; Hoyer, 1991). GR127935 is a 5-HT1B/1D antagonist with a high affinity for 5-HT1B (Starkey and Skingle, 1994). Overall, CGS12066B is a 5-HT1B/1D agonist, RU24969 is a 5-HT1B/1A agonist, and GR127935 is a 5-HT1B/1D antagonist, all with a greater affinity for 5-HT1B receptors. At first glance, when comparing the available 5-HT1B research on RRBs, the results appear conflicting but are more consistent when considering the various receptor targets these compounds can bind. The findings utilizing CGS12066B and GR127935 indicate that selectively increasing 5-HT1B/1D receptor activation induces RRBs, while selectively decreasing 5-HT1B/1D receptor activation reduces RRBs. To our knowledge, there are no studies that examine the effects that 5-HT1D receptors have on RRBs.

## 5-Ht2a

Serotonin 2A receptors are widely spread throughout the CNS with high concentrations in the frontal cortex, limbic system, claustrum, and basal ganglia (Xu and Pandey, 2000; Hoyer et al., 2002). Consistent with their high density in these areas, 5-HT2A receptors are involved in executive functions, and are the primary binding site for psychedelics and atypical antipsychotic medications (Fiorella et al., 1995; Vollenweider et al., 1998; Meltzer, 1999). Interestingly, the atypical antipsychotics risperidone and aripiprazole are the only FDA-approved medications for ASD. Although these drugs are only approved for reducing irritability in ASD individuals, risperidone has been found to alleviate behavioral deficits in the BTBR mouse model of ASD (Amodeo et al., 2014). Compared to other serotonin receptor targets, many more studies have examined how 5-HT2A receptor modulation impacts RRB expression. While these findings can be mixed, a general trend in the literature suggests that increased 5-HT2A activation increases RRB expression, whereas blockade generally decreases RRBs.

Although there is a trend in the literature showing that 5-HT2A activation increases head-twitch behaviors, these behaviors are more associated with hallucinogenic effects and were not included in our review. However, some studies demonstrate that 5-HT2A agonists also increase stereotypic behaviors. DOI, a 5-HT2 agonist with a high affinity for 5-HT2A receptors, was utilized in almost all studies examining how 5-HT2A activation affects RRBs. DOI administration in male Sprague–Dawley rats increased stereotypic gnawing, forepaw tapping, and skin-jerk behaviors (Gaggi et al., 1997; Wettstein et al., 1999). In male Wistar rats, DOI increased wet-dog shake behaviors (Kozuru et al., 2000) and other stereotypic motor behaviors (Hongyan et al., 2017a, b). All studies mentioned demonstrating an increase in RRBs following 5-HT2A activation, employed acute administration. One study found no significant impact of chronic 5-HT2A activation on marble burying behavior (Tsybko et al., 2020). Administration of three separate 5-HT2A agonists 25CN-NBOH, TCB2, and DOI over 14 days in C57Bl/6 male mice, did not produce significant changes in marble burying behavior. In contrast to findings demonstrating increased RRBs following 5-HT2A activation, Scalzitti et al. (1999) found that DOI pretreatment rescued repetitive self-grooming induced by the dopamine D1 receptor agonist SKF 38393 in male Sprague–Dawley rats. Odland et al. (2021b) found that DOI and 25CN-NBOH reduced marble burying in male C57BL/6J mice. Odland et al. (2021a) found similar reductions in marble burying in female NMRI mice treated with DOI. Interestingly, the 5-HT2A antagonist M100907 attenuated the decreased digging rates produced by DOI treatment but had no significant impact on marble burying when administered alone.

There are also mixed effects of 5-HT2A antagonism on RRBs within the literature, however, there is more evidence suggesting decreases in RRBs following 5-HT2A antagonism. Amodeo et al. (2016, 2017) examined the impact of 5-HT2A antagonism in the BTBR mouse model of autism. When administered systemically, the 5-HT2A antagonist M100907 (0.1 mg/kg) decreased elevated grooming behavior in BTBR mice. A follow up study showed that micro infusions of M100907 in the dorsomedial striatum and orbitofrontal cortex had opposing effects. Specifically, intra-OFC micro infusions potentiated elevated grooming, whereas intra-dorsomedial striatum micro infusions decreased grooming. In wild type mice, M100907 has been recently shown to attenuate LSD-induced elevated grooming (Rodriguiz et al., 2021). Contrastingly, Bruins Slot et al. (2008) did not observe a significant effect of M100907 on marble burying, whereas, the 5-HT2 antagonist ritanserin reduced marble burying. In the Scalzitti et al. (1999) study, chronic pretreatment with mianserin, another 5-HT2 antagonist, did not impact SKF 38393-induced repetitive self-grooming. Although mianserin is an overall 5-HT2 antagonist, chronic administration was previously shown to downregulate 5-HT2A receptors (Hensler and Truett, 1998). The differences in effects observed in 5-HT2A blockade may depend on acute compared to repeated administration, as well as the binding selectivity of the different compounds used.

Studies examining the impact of 5-HT2A modulation on RRBs are mixed, however, the differences in administration could account for the inconsistencies. The two studies that employed chronic administration did not observe significant impacts on RRBs, even though Tsybko et al. (2020) employed a 5-HT2A agonist and Scalzitti et al. (1999) tested a 5-HT2A antagonist. It is also important to consider differences in the RRB models used. For example, almost all studies employed drug-induced models of RRBs. However, Amodeo et al. (2016, 2017) used the BTBR mouse model of ASD, which exhibits characteristic behaviors analogous to RRBs in humans. There are possibly differential impacts of 5-HT2A modulation on RRBs when employed in a drug-induced model compared to a disorder model. Additionally, there are further differences within the drug-induced models. Scalzitti et al. (1999) was the only study to utilize a dopaminergic drug to produce RRBs. When used as a pretreatment, DOI attenuated elevated grooming induced by the dopamine D1 activation, however, most other studies have demonstrated that DOI typically produces RRBs, when administered alone. There may have been specific dopaminergic impacts on RRBs that should be explored in future studies. This section highlights a need for further studies examining the effects of chronic 5-HT2A modulation on RRBs, especially considering potential therapeutics for RRBs would likely be taken chronically.

## 5-Ht2b

5-HT2B is distributed in both CNS and PNS. Although these receptors are most abundant in the liver and kidney, they are also present throughout the brain, pancreas, and spleen (Bonhaus et al., 1995). To our knowledge, only two studies have explored the impact of 5-HT2B modulation on RRB expression. Kennett et al. (1997) examined the effects of 5-HT2B agonist BW723C86 on repetitive self-grooming in Sprague Dawley rats. They initially found that 1 and 10 μg of BW723C86 when infused into the right lateral ventricle had no effect on grooming. However, when administered subcutaneously and systematically at 10 and 20 (mg/kg), grooming frequency and duration decreased, indicating that these effects may be through peripheral systems. Effects of 5-HT2B antagonist LY266097 have also been tested on marble burying behavior in NMRI mice (Bruins Slot et al., 2008). LY266097 did not impact marble burying when administered intraperitoneally between 0.16 and 2.5 (mg/kg). Some evidence suggests 5-HT2B activation impacts the expression of RRBs, but this receptor target needs further examination.

## 5-Ht2c

5-HT2C receptors are distributed throughout the CNS, with high densities found in the choroid plexus. These receptors are primarily found throughout areas of the limbic system, specifically concentrated in the hippocampus, hypothalamus, septum, and neocortex. 5-HT2C is also expressed in areas important for motor actions, including the substantia nigra and globus pallidus (Hensler, 2012). 5-HT2C receptors are likely involved in several neurobiological processes including modulation of monoaminergic transmission, mood, and motor behavior (Chanrion et al., 2008). There is substantial evidence that this receptor is highly related to the expression of compulsive-like behaviors, defined as repetitious and ritualistic, although results are variable across studies (Flaisher-Grinberg et al., 2008).

Namely, mCPP, a 5-HT2C agonist, which in humans, has been observed to exacerbate symptoms of OCD in untreated patients (Gross-Isseroff et al., 2004) and in rodent RRBs (Reimer et al., 2018). Most studies exploring mCPP have observed increases in stereotypic behavior, commonly evaluated through repetitive grooming and marble burying and particularly at higher doses of 1 and 3 mg/kg (Bhutada et al., 2013; Wright and Rodgers, 2014). Moreover, Kantor et al. (2000) observed self-grooming behavior during a social interaction paradigm, finding that administration of mCPP in three separate rat strains enhanced self-grooming. Both Graf et al. (2003) and Bagdy et al. (2001) examined the effects of mCPP, which in both studies demonstrated increased grooming behaviors. Interestingly, there are several studies that have observed a decrease in these stereotypic behaviors with the upregulation of the 5-HT2C receptor. Halford et al. (1997) examined how increased activation of 5-HT2C receptors with the compound MK212, lead to an overall decrease in grooming behavior. Other 5-HT2C receptor agonists, Ro600175, Ro600332, and CPD1 similarly reduced marble burying behavior (Martin et al., 1998; Rodriguez et al., 2017). Together suggesting that increases in 5-HT2C receptor activation reduced rates of RRBs.

Previous studies suggest that 5-HT2C receptor antagonism does not increase stereotypic behaviors. Namely, Odland et al. (2021a) assessed how the 5-HT2C receptor contributes to marble burying behavior and found no differences with the 5-HT2C antagonist, SB242084 alone, compared to vehicle-treated mice. Conversely, Bagdy et al. (2001) observed a dose-dependent reduction in repetitive grooming with the administration of 5-HT2C antagonist SB242084, alone. Similar effects were found in Graf et al. (2003) who observed pre-treatment of SB242084 was able to effectively reverse the effects of grooming induced by mCPP. Together these findings suggest that 5-HT2C downregulation reduces RRB expression, and this is most evident when RRBs are pharmacologically induced.

Although studies on 5-HT2C receptor modulation are not consistent, there is evidence that this receptor is highly involved in behaviors relating to grooming and marble burying. The agonistic effects of 5-HT2C appear to vary depending on the specific 5-HT2C compound administered. In all instances, mCPP has been shown to increase RRBs, whereas the other 5-HT2C agonists (MK212, Ro600175, Ro600332, and CPD1) are shown to decrease RRBs. On the surface, these differences may be attributed to the secondary 5-HT receptor targets that these drugs show an affinity for. These drugs are vastly more selective for 5-HT2C, yet still show affinity for other 5-HT receptors, such as 5-HT2B and 5-HT2A. As for the 5-HT2C antagonist, most instances show that blocking 5-HT2C activation has no effect on RRBs. The instances where 5-HT2C antagonists decreased RRBs, were only observed in Bagdy et al. (2001) when grooming was measured during a social interaction anxiety test and Graf et al. (2003) only after animals were grooming was induced from mCPP. Considering these differences, 5-HT2C receptor activation can modulate RRBs in either direction depending on the 5-HT2C drug used. Blocking 5-HT2C activation only seems to show therapeutic capabilities when RRBs are pharmacologically induced.

## 5-Ht3

Of the several 5-HT3 (5-HT3A/B/C/D/E), only 5-HT3A/B/C are found in the brain (Yamada et al., 2006). 5-HT3 receptors are expressed in the hippocampus, the amygdala, and the cerebral cortex (Morales et al., 1996; Hájos et al., 2000). Until recently, only one study has considered the effects that 5-HT3 on measures of RRBs. Gillies et al. (1996) examined the affect of selective 5-HT3 agonist 2-methyl-5-H treatment on self-grooming in Sprague–Dawley rats. Although grooming was not the focus of this study, the authors do briefly mention that grooming was not affected by 2-methyl-5-HT exposure. More recently, Huang et al. (2021) employed a Htr3a knockout mouse and measured RRB expression. Compared to the wild type, Htr3a KO mice displayed elevated self-grooming rates, suggesting that reduced 5-HT3A receptors development may lead to increased repetitive grooming behavior.

## 5-Ht6

Similar to previously discussed 5-HT receptors, 5-HT6 is found in high concentrations in the hippocampus, amygdala, and the cerebral cortex. 5-HT6 receptors are also expressed in the nucleus accumbens, dentate gyrus, olfactory tubercles, and entorhinal cortex (Gérard et al., 1996; Hirst et al., 2003; de Assis Brasil et al., 2019). Two studies have examined how 5-HT6 affects RRB expression, including the 5-HT6 agonist WAY208466 and 5-HT6 antagonist BGC20761. Pereira et al. (2015) measured rates of self-grooming in C57BL/6J mice during the training and test sessions of a passive avoidance task. Treatment with the 5-HT6 agonist WAY208466 elevated grooming rates in C57BL/6J mice. Amodeo et al. (2021) measured the effects of the 5-HT6 antagonist BGC2076 in C57BL/6J and BTBR mice, a strain that expresses high rates of grooming behavior. Both male and female BTBR mice displayed elevated grooming behaviors compared to C57BL/6J mice. BGC20761 had no effect on C57BL/6J normal mice but decreased the elevated grooming behaviors observed in BTBR mice. In the same study, C58/J inbred mouse strain that expresses elevated repetitive spontaneous jumping, another RRB, was also treated with BGC20761. Unlike the repetitive grooming behaviors observed in BTBR mice, BGC207 did not attenuate the elevated jumping and flipping behaviors observed in C58 mice. Together these studies suggest that increasing 5-HT6 receptor activity increases RRB expression while blocking 5-HT6 activity decreases RRBs. However, due to the limited amount of data, these results appear to be task and strain-specific.

## 5-Ht7

The most recently identified serotonin receptor, 5-HT7 has been shown to be involved in circadian regulation, thermoregulation, mood, and the gastrointestinal systems (Glass et al., 2003; Hedlund et al., 2004). There are also high densities of 5-HT7 receptors in the thalamus, dentate gyrus of the hippocampus, with lower concentrations found in the cortex, septum, hypothalamus, and CA1 and CA2 regions of the hippocampus (Hannon and Hoyer, 2008). While there is limited data available on the 5-HT7 receptor, studies have found that both agonists and antagonists decrease RRB expression. The 5-HT7 agonist, (+)-5-FPT, eliminated idiopathic stereotypic jumping in C58/J mice and decreased self-grooming in C57BL/6J mice (Canal et al., 2015). Hedlund and Sutcliffe (2007) found that the inactivation of 5-HT7 receptors in OCD model mice can attenuate repetitive burying behavior. This study also found that 5-HT7 KO mice buried fewer marbles than 5-HT7/+ mice. Although the 5-HT7 antagonist SB269970 did not affect the 5-HT7−/− mice, it decreased marble burying in 5-HT7+/+ mice. Together, these studies suggest that either increasing or blocking 5-HT7 activity can reduce RRBs.

## Conclusion

The current review highlights how much of the research seems to focus on select receptors, such as the 5-HT1A, 5-HT2A, and 5-HT2C receptors and it is difficult to determine why this is. One possibility is that these receptors have a greater effect on RRB expression, but this is difficult to determine from the current findings. It is also possible that the compounds that bind to these receptors are readily available, thus making it easier to attain and test. Alternatively, because there is a demand for these specific receptor compounds, their affinity for those specific receptors has been refined, compared to the other less examined 5-HT receptor targets.

Alleviating RRB expression may require drugs that have a broader receptor profile, although this is not the focus of the current review. The current review provides a rationale for developing new compounds that take a multiple receptor approach to attenuating RRBs. To do this we need to design more studies that use this approach. The current findings begin to shine a light on the impact of specific serotonin receptor modulation on RRBs and highlight some of the contradictory findings commonly found with these receptor targets. Interestingly, the newer identified 5-HT6 and 5-HT7 receptors, although few studies have been conducted, show evidence of being effective in attenuating RRBs. This is encouraging in the search to find new therapeutic treatments. In sum, the hope is that these findings will produce novel therapeutics for neuropsychiatric disorders afflicted by repetitive behaviors.

## Author Contributions

BA, CC, CM, SL, and DA were involved in writing and design of the review. All authors contributed to the article and approved the submitted version.
